# Adapting a Text Messaging Intervention to Improve Diabetes Medication Adherence in a Spanish-Speaking Population: Qualitative Study

**DOI:** 10.2196/66668

**Published:** 2025-05-01

**Authors:** Jacqueline Seiglie, Seth Tobolsky, Ricaurte Crespo Trevino, Lluvia Cordova, Sara Cromer, A Enrique Caballero, Margarita Alegria, J Jaime Miranda, Deborah Wexler, Lindsay Mayberry

**Affiliations:** 1 Department of Medicine, Harvard Medical School, Boston, MA, United States; 2 Diabetes Unit, Massachusetts General Hospital, 50 Staniford St, Suite 340, Boston, MA, 02114, United States, 1 6177268722, 1 6177248534; 3 Mass General Brigham Salem Hospital, Salem, MA, United States; 4 Division of Endocrinology, Diabetes and Hypertension, Brigham and Women’s Hospital, Boston, MA, United States; 5 Disparities Research Unit, Department of Medicine, Massachusetts General Hospital, Boston, MA, United States; 6 Departments of Medicine & Psychiatry, Harvard Medical School, Boston, MA, United States; 7 Sydney School of Public Health, Faculty of Medicine and Health, University of Sydney, Sydney, Camperdown, Australia; 8 Vanderbilt University Medical Center, Nashville, TN, United States

**Keywords:** diabetes, diabetes mellitus, DM, type 2 diabetes, T2DM, type 1 diabetes, endocrinology, medication adherence, adherence, Latino adults, stakeholder engagement, text messaging, messaging, intervention, multiphase, nonadherence, glycated hemoglobin, HbA1c, Latino, stakeholder, medication, Spanish-speaking, population

## Abstract

**Background:**

Latino adults with type 2 diabetes (T2D) have higher rates of diabetes medication nonadherence than non-Hispanic White adults. REACH (Rapid Encouragement/Education And Communications for Health) is a text message platform based on the information-motivation-behavioral skills model that addresses barriers to adherence and was shown to improve adherence and glycated hemoglobin (HbA1c) levels, but it is only available in English.

**Objective:**

This study aimed to report the multiphase, stakeholder-driven adaptation of the REACH barriers to diabetes medication adherence content to a Latino population (REACH-Español).

**Methods:**

This was a qualitative study using focus groups. We identified potentially eligible patients (≥18 y old, Latino ethnicity, Spanish-language preference, and T2D diagnosis) using a Mass General Brigham Hospital query. Eligible patients were invited to participate in a focus group conducted in Spanish between April 13 and November 9, 2023. A total of 5 focus groups were conducted. Focus groups 1‐3 centered on ranking 40 barriers to diabetes medication adherence (derived from REACH and the extant literature), whereas focus groups 4‐5 centered on translation and cultural modifications of the original SMS text message content associated with each of the REACH barriers. Barriers were mapped onto information-motivation-behavioral constructs. We used descriptive statistics to summarize participant characteristics. Focus groups were audio-recorded, professionally transcribed, and analyzed with thematic content analysis using NVivo (Lumivero).

**Results:**

In total, 22 participants attended the focus groups. The mean (SD) age was 63.2 (11) years, 55% (n=10/22) were female, and the mean HbA1c level was 8.5%. All participants were born in Latin America or the Caribbean and spoke Spanish as their preferred language, and 54.5% (12/22) had completed middle-school education or less. Among the top 10 ranked barriers, 50% (n=5) corresponded to information, 20% (n=2) to social motivation, 20% (n=2) to behavioral skills, and 10% (n=1) to personal motivation. Personal motivation barriers (medication burden and fear of side effects) and behavioral skills (forgetting to take medication) emerged as important themes in the focus groups.

**Conclusions:**

A stakeholder-driven approach to intervention adaptation identified and prioritized relevant barriers to diabetes medication adherence among Latino adults with T2D and facilitated the adaptation of the REACH platform to a Spanish-speaking population.

## Introduction

Suboptimal medication adherence, generally defined as taking less than 80% of prescribed medications [1], is reported by nearly 50% of adults with type 2 diabetes (T2D) [2,3] and is associated with poor glycemic management [1,2,4] and higher risk of all-cause mortality [5]. Rates of suboptimal diabetes medication adherence are higher in racial and ethnic minority populations [6,7], particularly among individuals with lower English language proficiency [8]. An estimated 60% of Latino adults with limited English language proficiency report nonadherence to diabetes medications, compared with 52% of Latino adults with English proficiency and 38% of non-Hispanic White individuals [8]. Unique barriers to diabetes medication adherence have been reported by Latino adults with T2D [8-18], including negative perceptions regarding diabetes medication use (particularly insulin) [9,12,18], misunderstanding of the ongoing need for glucose-lowering pharmacotherapy once glycated hemoglobin (HbA1c) has improved [17], and challenges with regimen complexity even with the use of a Spanish-language interpreter [8,19]. While interventions that address patient-reported barriers to diabetes medication adherence can improve glycemic management [20,21], interventions that address barriers to diabetes medication adherence of relevance to Latino adults with T2D are lacking.

Among numerous behavioral theories that explain medication adherence behavior [22], the information-motivation-behavioral skills (IMB) model is an important theory-based model for conceptualizing and addressing domains associated with diabetes medication adherence [23,24]. The IMB model is built on three key components that influence behavior change, that are (1) information (ie, accurate knowledge about the disease and medications), (2) motivation (ie, beliefs and attitudes plus social support and social norms), and (3) behavioral skills (ie, objective and perceived abilities) [23,25]. When applied to diabetes medication adherence behavior, the IMB components allow for the identification of barriers to medication adherence in each domain [25]. Several interventions that have incorporated this model to address self-reported barriers to medication adherence have shown improvements in diabetes medication adherence and glycemic management among adults with T2D, including among Latino adults [21,23]. However, patient-centered tools for Latino adults with T2D based on the IMB model that are scalable and that do not rely on in-person educational sessions, which can be resource-intensive, do not yet exist.

REACH (Rapid Encouragement/Education And Communications for Health) is an innovative text message tool based on the IMB model that delivers text message content tailored to individual patient-reported barriers to diabetes medication adherence [21,26]. REACH delivers daily diabetes self-management 1-way texts, daily 2-way texts that ask about medication adherence, and weekly texts that provide adherence feedback and encouragement based on responses to 2-way texts. REACH was shown to improve diabetes medication adherence (by an average of one-third to one-half day per week over 12 months), reduce barriers to diabetes medication adherence (including cost-related barriers), improve dietary behavior and physical activity, and significantly reduce HbA1c at 6 months compared with usual care (0.74% reduction among patients with baseline HbA1c≥8.5%) [21]. However, despite its efficacy, scalability, and high participant engagement [27], REACH has not been adapted to other contexts. REACH could serve as an important clinical tool to address barriers to diabetes medication adherence among Latino adults with T2D. In this study, we used a multistage, stakeholder-driven, iterative method of language adaptation to adapt the original REACH barrier content to a Latino, Spanish-speaking population with T2D, focusing on identifying barriers to diabetes medication adherence of relevance to this population.

## Methods

### Study Design, Setting, and Participant Eligibility

We conducted a qualitative study by carrying out focus groups at the Massachusetts General Hospital (MGH) Chelsea HealthCare Center, one of the MGH Community Health Care Centers that serve a patient population in the greater Boston area that is majority Latino or Spanish-speaking [28]. Eligible participants had a T2D diagnosis (based on diagnostic codes, confirmed with electronic health record review), were ≥18 years old, were taking at least 1 glucose-lowering medication, and reported Spanish as their preferred written and spoken language. We excluded patients with auditory or communication difficulties.

### Participant Recruitment

We searched the MGH Research Patient Data Registry to identify individuals who received care at MGH within the previous 2 years and who were ≥18 years old, had a diagnosis of T2D, and had registered Spanish as their preferred language. A total of 3709 patients met this criteria, of whom 500 lived in the city of Chelsea. We mailed out opt-out letters to 350 of these participants (selected randomly), followed by telephone calls to screen for the eligibility criteria described above. Among 30 eligible patients willing to participate in the study, 22 unique participants attended one of the focus groups 1‐5. A total of 10 participants who attended focus groups 1‐3 also attended either focus group 4 or 5. The reason for participation in multiple focus groups was to engage patients in the iterative process of content adaptation, which took place in two steps: (1) identification of barriers to diabetes medication adherence of relevance to the study population (focus groups 1‐3) and (2) adaptation of SMS text message content addressing the relevant barriers. Enrollment occurred between March and November of 2023. Given that the original REACH content was created with input from communication experts who edited the text messages to be readable and understandable (ie, written at the sixth-grade reading level or below) [26], the adaptation of REACH barrier content was also guided by an emphasis on reading appropriateness for the study population. Thus, for eligible patients interested in participating in the study, we conducted a health literacy screening to tailor the focus group activities to the literacy level of the study population. We used the 1-item Brief Health Literacy Screening Tool (BRIEF) questionnaire (“How confident are you filling out medical forms by yourself?”), which has been validated for Spanish-speaking populations as a quick assessment of health literacy [29].

### Ethical Considerations

Participants received US $50 for focus group attendance. The Mass General Brigham Institutional Review Board approved all aspects of the study (protocol 2022P002678). At the beginning of each focus group, participants received an IRB-approved information sheet, which outlined the objectives of the study, procedures, benefits and risks, confidentiality, as well as an emphasis on voluntary participation. All participants agreed to participate in the focus groups.

### Measures

At the start of each focus group, participants were asked to fill out a sociodemographic and diabetes history questionnaire. The data that were collected through this questionnaire were age, sex, ethnicity, country of birth, preferred language, educational attainment, household income, health insurance, and diabetes history (age at diagnosis, diabetes medications, and complications).

### REACH Barriers to Diabetes Medication Adherence

The development of the REACH barrier content has been described in studies by Nelson et al [25,26]. The REACH study team conducted a thorough review of published studies describing patient-reported barriers to adherence to glucose-lowering medications. The literature review yielded 68 barriers to taking oral glucose-lowering medications and 7 barriers to taking insulin; similar barriers were collapsed, resulting in 36 adherence barriers [25]. A 2-step interactive procedure was subsequently used to rank these barriers among adults with T2D. Each of the 36 barriers was mapped to the IMB constructs, and content experts drew on identified studies to develop SMS text messages addressing each barrier [26]. For instance, the barrier “My daily medicine routine is too complicated to keep track of” generated 21 SMS text messages to address this concern, such as “to help keep things simple, take your medications at the same time you’re doing something else, like brushing your teeth or eating meals.”

### Adaptation Methodology

#### Description

**Figure 1.**
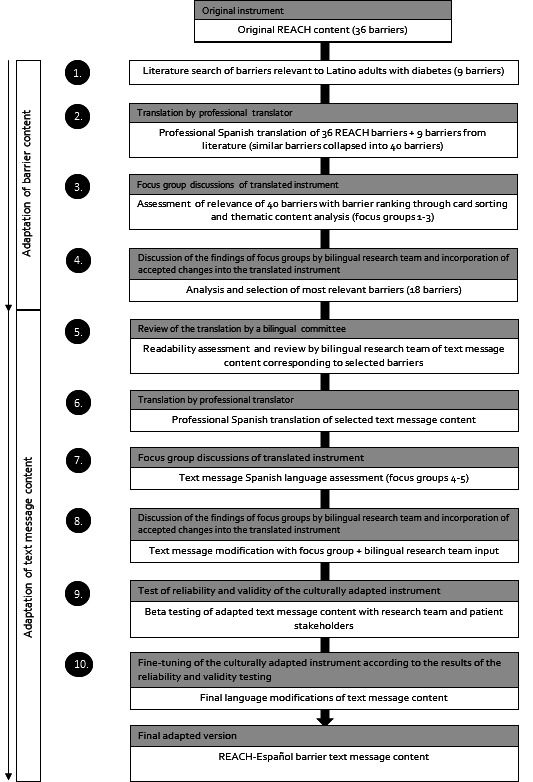
Adaptation and development of REACH-Español text message content on barriers to diabetes medication adherence (gray boxes outline original adaptation framework proposed by Matias-Carrelo et al [30]; corresponding modifications are shown in the white boxes). REACH: Rapid Encouragement/Education And Communications for Health.

To ensure that the REACH-Español barrier content was culturally and linguistically appropriate for the target population, we were guided by the multistage, iterative method for cross-cultural adaptation proposed by Matías-Carrelo et al [30] This multistage method relies on the linguistic review of the translated instrument by incorporating input from multiple stakeholders, including both a multinational bilingual committee composed of bilingual (English-Spanish) researchers familiar with the instrument and stakeholders from the populations being studied. This adaptation methodology aims to maintain the content, semantic, and technical equivalence. It is superior to the translation and back-translation method, which may not yield cultural equivalency of the content being translated, nor capture key sociocultural factors [30]. In this study, two groups of bilingual stakeholders reviewed the SMS text message content being translated (1) a bilingual research team and (2) focus group participants. Figure 1 summarizes the 10-step adaptation process used in this study (the Matías-Carrelo et al [30] framework is shown in gray and the corresponding adaptations in white).

#### Literature Review of Barriers and Translation

We conducted a systematic review of published studies describing patient-reported barriers to diabetes medication adherence in Spanish-speaking adults with T2D. We identified an additional 9 barriers relevant to Spanish-speaking adults that were not included in the original REACH barrier list (Multimedia Appendix 1). We collapsed similar barriers, resulting in 40 barriers to diabetes medication adherence with potential relevance to Spanish-speaking adults with T2D (Multimedia Appendix 2).

#### Barrier Translation

The 40 barriers’ names were professionally translated into Spanish (Multimedia Appendix 1).

#### Barrier Assessment

To assess the relevance (and irrelevance) of the 40 barriers to diabetes medication adherence among Latino adults with T2D, we conducted 3 focus groups. Our screening for health literacy at recruitment showed that 33% of participants interested in attending one of the focus groups had low health literacy (“not at all” confident in filling out medical forms by yourself). Therefore, we tailored the focus group activities to a lower health literacy level by focusing on nonwritten activities and using visual props and cues to guide the discussion (Multimedia Appendix 3). We used a 2-step interactive process adapted from the original REACH barrier development process [25]. In the first activity, we encouraged an open discussion by asking participants to think about “things that make taking diabetes medications difficult” and to share these with the group; we wrote each item on a whiteboard as the discussion progressed. In the second activity, we asked participants to rank each of the 40 barriers to diabetes medication adherence referenced earlier, according to personal relevance. To make the activity easy to follow, we used a “traffic light” model of 3 colored envelopes and asked participants to place each written barrier name (eg, “I worry that taking diabetes medicines for a long time will be bad for me”) into a folder, based on its relevance to them (Multimedia Appendix 3): red (never), yellow (sometimes), or green (always). Focus groups were audio-recorded and professionally transcribed in Spanish.

#### Barrier Analysis and Selection

Upon completion of the barrier ranking activity, barriers that were placed in the red “never” envelope received a score of 1, those placed in the yellow “sometimes” folder received a 2, and barriers placed in the green “always” folder received a 3. We then calculated the proportion of participants that reported each barrier as sometimes or always (Multimedia Appendix 4). We retained 23 barriers that were reported as relevant ≥33% of participants (mean proportion for all barriers was 32%) plus 1 barrier (regimen complexity) that emerged as an important theme in the focus groups. We then combined barriers with similar content (19 barriers) and removed 1 barrier (“I think brand name medicine works better than generic medicine”) because of some recent recalls of generic glucose-lowering medications which may have affected perceptions at the time [31], resulting in 18 final barriers for REACH-Español.

#### Review of Existing Barrier Text Message Content

To adapt the text message content corresponding to each barrier in a Spanish-speaking population, we selected the most relevant barriers that were identified in focus groups 1‐3. We reviewed the original REACH text message English content corresponding to each of the selected barriers and conducted a readability check of each text message using the Flesch-Kincaid readability grade level test, ensuring that the language used was grade 6 or lower.

#### Text Message Content Translation

The text message content corresponding to each of the 18 barriers was professionally translated into Spanish.

#### Text Message Spanish Language Assessment

To ensure that the Spanish text message content was culturally and linguistically appropriate for the target population, we invited the same group of participants that attended focus groups 1‐3 to attend 1 of 2 additional focus groups (focus groups 4‐5). To solicit participant feedback in the focus groups, we projected each text message on a Microsoft PowerPoint presentation and gave each participant a green (“Yes) and a red (“No”) paddle (Multimedia Appendix 5). We asked participants to approve or disapprove of messaging according to ease of understanding by raising the paddle when each text message was projected and read aloud. We discussed messages that received “No” votes.

#### Text Message Content Modification

Following the completion of focus groups 4‐5, we excluded text messages not well-received by participants (not relevant, tone did not resonate). Then, our bilingual research team modified specific language based on participant feedback, with a focus on selecting language that was more broadly understood by Spanish-speaking participants from diverse geographic and cultural backgrounds with distinct dialects. Focus groups were audio-recorded and professionally transcribed in Spanish.

#### Beta Testing

The revised text message content was sent to our digital health partner, MEMOTEXT, which will deliver the text messages to participants in the pilot study of REACH-Español. To ensure that the text message content delivery worked as intended and to subject the content to one more round of language modification, we conducted beta testing of the text message content. Beta testing entailed having the research team members as well as 3 patient stakeholders (participants who attended focus groups 4 or 5) receive and interact with the REACH-Español text message content over the course of 4 weeks. All members of the beta testing process provided input on a weekly basis on either technical or linguistic aspects that required modification. Patient stakeholders provided their impression of the program via an exit interview.

#### Fine-Tuning

Throughout the beta testing process, we identified several technical issues, mostly pertaining to the sent time of the text messages (ie, too close to bedtime, welcome text message sent after content message). MEMOTEXT revised the platform throughout the beta testing process based on user feedback. After all user input was incorporated, a final version of the REACH-Español text message content was created.

### Analysis

We used descriptive statistics (means, SDs, and proportions) to summarize participant characteristics. For the qualitative analysis, an “open-coding” approach (inductive) was used first to identify themes in the transcripts (in Spanish) from focus groups 1‐3. Themes were then categorized as barriers and facilitators to diabetes medication adherence. Second, we used deductive coding to map the codes into the IMB construct. Coding was conducted by native Spanish-speaking research team members. Analyses were conducted using Microsoft Excel and NVivo 14 (Lumivero).

## Results

### Focus Group Participants

A total of 22 participants attended focus groups 1‐5. The first 3 groups were attended by 18 participants; 10 of these participants also attended either focus group 4 or 5. Table 1 summarizes the demographic and clinical characteristics of the study participants. The mean age was 63.2 (SD 11) years, 55% (10/22) were female, 59% (13/22) were categorized as having high health literacy (based on the 1-item BRIEF questionnaire), although 55% (12/22) reported an educational attainment of elementary or middle school only. Over two-thirds (16/22 73%) of participants were born outside of the United States, largely in Central America. Participants had a mean (SD) diabetes duration of 20.3 (12) years. In addition, 59% (13/22) of participants reported insulin use.

**Table 1.** Characteristics of the focus group population.

Characteristic	Overall (n=22)
Age (y), mean (SD)	63.2 (11)
Sex (female), n (%)	10 (55)
HbA_1c_, mean (SD)	8.5 (1)
HbA_1c_<7%, n (%)	2 (9)
HbA_1c_>7%, n (%)	20 (91)
Age at diabetes diagnosis (y), mean (SD)	44.2 (13)
Diabetes duration (y), mean (SD)	20.3 (12)
Number of diabetes medications, mean (SD)	2.9 (1)
Insulin use, n (%)	13 (59)
Health literacy quintile, n (%)	
1 (lowest)	3 (14)
2	1 (4)
3	2 (9)
4	3 (14)
5 (highest)	13 (59)
Educational attainment, n (%)	
Elementary or middle school	12 (55)
Some secondary schooling	4 (18)
High school or GED	4 (18)
Some university	2 (9)
Place of birth, n (%)	
Colombia	1 (5)
Dominican Republic	1 (5)
El Salvador	5 (23)
Guatemala	5 (23)
Honduras	3 (14)
Peru	1 (5)
Puerto Rico	6 (27)

### Barrier Selection

**Figure 2.**
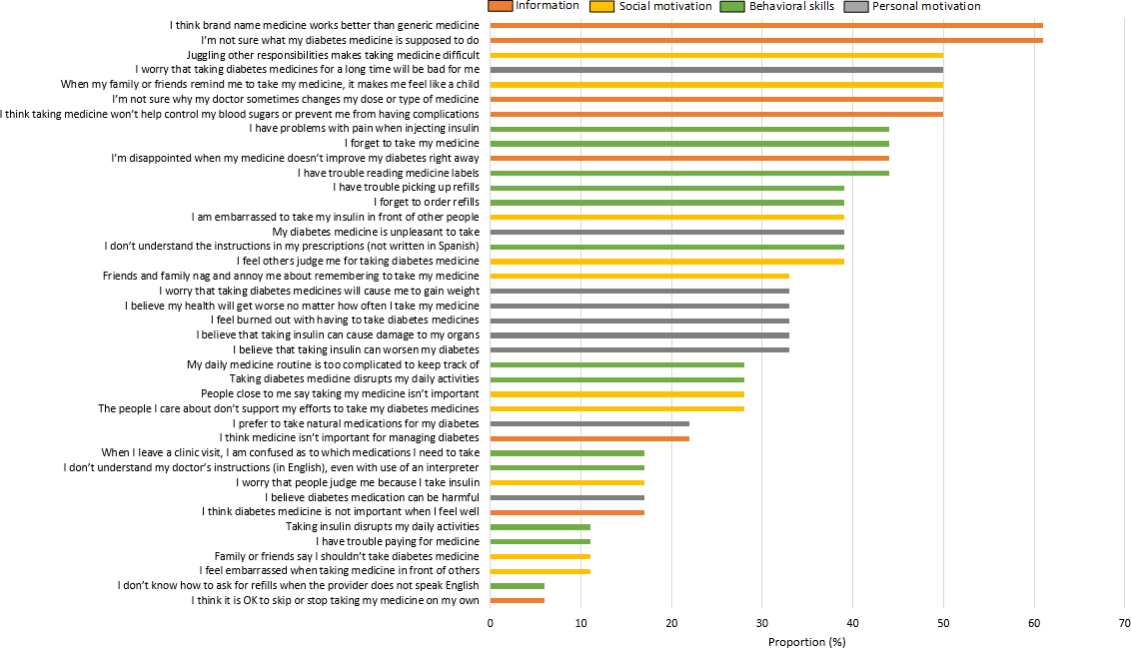
Proportion of participants who reported each diabetes medication adherence barrier as relevant sometimes or always (barriers were shortened to improve readability, please refer to Multimedia Appendix 4 for full barrier wording).

Multimedia Appendix 2 shows the list of 40 barriers to diabetes medication adherence that were assessed for relevance in focus groups 1‐3. Barriers are shown in order of higher to lower proportion of participants reporting each barrier as relevant sometimes or always. Figure 2 shows the list of 40 barriers, categorized into the IMB model and ranked according to the proportion of participants who reported the barrier as relevant sometimes or always. Among the top 10 barriers, 50% (n=5) corresponded to the information category, 20% (n=2) to the social motivation category, 20% (n=2) to behavioral skills, and 10% (n=1) to personal motivation.

### Barriers to Diabetes Medication Adherence Based on Thematic Analysis

Table 2 outlines the key themes that emerged in focus groups 1‐3 based on participant quotes, grouped into barriers to diabetes medication adherence, and mapped into the IMB model. In total, 5 themes emerged in response to the question “What are some difficulties that come to mind when taking your diabetes medications?” In order of relevance (higher or lower number of times mentioned), most participants agreed that medication burden was one of the most important barriers to diabetes medication adherence, followed by fear of side effects or of new medication, difficulty or confusion with medication changes, forgetting to take medications, and frustration with diagnosis or lack or improvement.

**Table 2.** Diabetes medication adherence barriers reported by focus group participants (what are some difficulties that you encounter when taking your diabetes medications?).

Barriers to diabetes medication adherence: What are some difficulties that you encounter when taking your diabetes medications?			
Themes	Participant Quotes	IMB^a^ category	Existing REACH^b^ barrier
Medication burden			
Too many medications and exhaustion with taking medications daily	“It’s not difficult to take medications, it’s just that we are disgusted by having to take so many medications.” (Female, 62 y old, El Salvador) “I am exhausted with having to take medications every day.” (Female, 45 y old, Guatemala) “Sometimes I don’t want it….there is a rejection, a rejection [of the medication].” (Female, 45 y old, El Salvador)	Personal Motivation	I feel burned out with having to take diabetes medicines
Interferes with daily activities	“Yes, [it interferes] with daily chores…if the next dose is close enough, then I skip it and inject the following dose.”(Male, 63 y old, El Salvador). “Well, sometimes it’s very difficult to wake up, knowing that you won’t be drinking a cup of coffee with bread, but something else, to take the diabetes pills.” (Female, 74 y old, Puerto Rico)	Social Motivation	Taking diabetes medicine disrupts my daily activities/ Taking insulin disrupts my daily activities
Regimen complexity	“It’s very difficult because I take ten medications in the morning and eight at night.” (Male, 63 y old, El Salvador). “In my case, I have to take two pills in the morning and two at night…and the daytime ones, I can’t get used to taking them. I take all four at night.” (Male, 63 y old, El Salvador)	Behavioral skills	My daily medicine routine is too complicated to keep track of
Juggling other responsibilities	“I have difficulty taking the medications because I start working at 7AM, so if I am late for work I don’t take any medications until my break time, around 10am…and then the other medications I take at 9PM, when I come back from work.” (Female, 45 y old, Guatemala)	Social Motivation	Juggling other responsibilities makes medicine difficult
Fear of side effects or of new medications			
Low blood sugars	“And there are times when it [blood sugar] drops suddenly and it gives me anxiety to find something to eat because I feel like as he [other participant] mentioned, tremulous, it’s horrible.” (Male, 56 y old, Guatemala)	Personal Motivation	N/A^c^
Side effects	“Perhaps I will not feel it now but I will feel it [side effects] over time.” (Male, 65 y old, Guatemala)	Personal Motivation	I worry that taking diabetes medicines for a long time will be bad for me
New medications	“You are taking a risk, if you take the medication, it alleviates something but it will damage something else.” (Female, 63 y old, Puerto Rico)	Information	N/A
Organ damage	“Because you know that there are medications that damage the kidneys and then the doctor tells you, I’m sorry, it was the medication [that caused the kidney damage], so you live in fear.” (Female, 71 y old, Puerto Rico)	Personal Motivation	I believe diabetes medication can be harmful.
Difficulty or confusion with medication changes			
Medication changes	“I had a problem with the insulin because, I mean, my sugar was very high so the doctor said ‘we are going to do an experiment,’ they did an experiment with me that they put me until a certain level and that’s where it [the dose] remained.” (Male, 65 y old, Guatemala) “Because they have me like a guinea pig, like playing with me, making experiments…sincerely don’t know what to do.” (Male, 56 y old, Guatemala)	Information	I’m not sure why my doctor sometimes changes my dose or type of medicine
Medication instructions	“Í would leave [the clinic] and would write down the [medication] changes but then I would lose the paper…so I wouldn’t know if I had to put 35 [units] in the morning or 38 in the afternoon…and since I had a visit every 3 mo, I would suffer very much.” (Male, 77 y old, Guatemala)	Information	When I leave a clinic visit, I am confused as to which medications I need to take
Forget to take medications			
	“In reality, it’s not difficult to take the medications…the problem is that I forget to take the medications because of my age.” (Male, 77 y old, Guatemala) “Yes, I have one difficulty in taking my medications. That is, I am very forgetful.” (Male, 54 y old, El Salvador)	Behavioral skills	I forget to take my medicine
Frustration with diagnosis or lack of improvement			
Treatment doesn’t work	“At times I feel very frustrated because so many medications that you are taking and the sugar does not go down….that puts me depressed.” (Female, 62 y old, El Salvador)	Information	I’m disappointed when my medicine doesn’t improve my diabetes right away
Difficulty accepting diagnosis	“I have diabetes since the last two years. I come from a family with diabetes. I got divorced and in the process, the diabetes appeared. So, I started taking the metformin because I didn’t want to accept it. It was something very hard and I have difficulty accepting it.” (Female, 45 y old, Guatemala)	Personal motivation	N/A
Labile blood sugars	“The sugar went down but now I have a problem that the sugar went up, so there’s no balance, how to control this, sincerely it is very, very complicated for me and I don’t know what to do.” (Male, 56 y old, Guatemala)	Information	N/A

a IMB: information-motivation-behavioral skills model.

b REACH: Rapid Encouragement/Education And Communications for Health.

c N/A:not applicable

### Facilitators to Diabetes Medication Adherence Based on Thematic Analysis

Multimedia Appendix 6 shows the 5 themes that emerged in response to the question, “What motivates you to take your diabetes medications.” In order of relevance (higher to lower), fear as motivation was an important facilitator, followed by belief in treatment benefit, personal responsibility, and will to take medications, family support and medication organization*,* and getting used to the medication.

### Language Modification of Text Message Content

Focus groups 4‐5, which centered on eliciting feedback on the translated text message language content, generated rich discussion. A total of 111 text messages (related to barriers 1‐8) were reviewed in focus group 4 and 100 text messages (related to barriers 9‐18) were reviewed in focus group 5. Participants were generally enthusiastic about the text message content and provided feedback on wording that seemed confusing, too complex, or less likely to be understood by broad Spanish-speaking audiences. For instance, the word “comprimidos” was changed to “pastillas” and the preferred word for refills was “reposiciones” instead of “prescripciones.” In total, 12 messages were excluded since patients did not like the tone, felt that they were difficult to understand, or felt that they were not relevant. Some examples of these excluded text messages included “If taking your diabetes medications is unpleasant, try to push through. Remember that not taking them may make you feel worse” and “What advice would you give your friends who forget to refill their medications? Use those tips for yourself too!”

### Beta Testing Patient Stakeholder Input

In the final step of REACH-Español content adaptation, 3 patient participants who had attended one of the focus groups were invited to participate in the beta testing process over the course of 4 weeks. At the end of beta testing, the following patient stakeholder input was provided:

The texts are very important because when you are old, you know things from experience, but you forget things that are important. Like that the test strips need to be in a hermetically sealed box. The text messages are very helpful with remembering to take the medications. [Male, 77 years old, Guatemala]

The texts are helping to remind me to take my medications. When I look and I see the message [any of the messages], I run to take my medication. The texts are very clear. It’s really helping me. [Female, 45 years old, Guatemala]

The content is helpful. For example, learning about what food to eat to raise a low blood sugar. It’s helping to remind me to take my medications. I think this program is best for older people who have the time and attention to follow the advice. [Male, 63 years old, El Salvador]

## Discussion

### Principal Findings and Comparison With Previous Works

In this study, we outline the multistage process used to adapt the REACH text message content [26], which addresses patient-reported barriers to diabetes medication adherence, to a Latino population. The adaptation process centered on the theory-based IMB skills model for medication adherence and engagement of patient stakeholders to identify barriers to diabetes medication adherence with specific relevance to Latino adults with T2D (with Spanish-speaking preference). We found that among the top 10 most commonly reported barriers to diabetes medication adherence among Latino adults with diabetes, 50% (n=5) corresponded to information gaps, 20% (n=2) to social motivation, 20% (n=2) to behavioral skills, and 10% (n=1) to personal motivation. In contrast, our thematic analysis showed that personal motivation barriers (medication burden and fear of side effects) were the highest concern for focus group participants, followed by behavioral skills (forgetting to take medications), and information barriers (difficulty or confusion with medication changes and frustration with diagnosis or lack of improvement). These findings suggest that using the IMB model as a framework to identify barriers to diabetes medication adherence is useful in this population and that different qualitative approaches (open discussion vs ranking exercise) can provide complementary data to inform the adaptation process of tailored interventions.

By engaging focus group participants in an interactive barrier ranking exercise conducted through card sorting, we were also able to select highly relevant barriers to diabetes medication adherence and exclude those with lower salience from the study. Through this exercise, we found that the priority of some barriers differed in this population compared with the original REACH intervention. For instance, “I’m not sure what my diabetes medicine is supposed to do” was reported as relevant by 61% (11/18) of participants in this study, whereas this barrier was relevant only for 16% (38/237) of participants in the REACH development study [25]. Similarly, “I am not sure why my doctor sometimes changes my dose or type of medicine” was relevant to 50% (9/18) of participants in this study, whereas only 15% (36/237) of participants in the REACH development study reported that this barrier was relevant. These findings underscore the importance of stakeholder-driven adaptation approaches given that core intervention components (in this case, the list of barriers), differed across populations.

We also found that the iterative process of translating and modifying the original intervention content with stakeholder input (bilingual research team, focus group participants) enriched the adaptation process by incorporating language diversity and nuance. By eliciting input from participants through interactive focus group activities, we were able to capture both the thematic and linguistic or cultural relevance of the content being translated. For instance, we excluded certain text messages that did not resonate with the audience. We revised the language with modifications considered more broadly understood across Spanish-speaking Latin American and Caribbean populations. Choosing language wording with a broader reach was particularly important in this study given the heterogeneity of the Spanish language across Latin America and the Caribbean. Our observations are consistent with previous studies that have highlighted the importance of engaging bilingual stakeholders (including patients) throughout the language adaptation process [30,32,33].

Although barriers to diabetes medication adherence have been previously reported in the Latino population [8,10,11,17,19,34], most studies have not used a theoretical framework to identify such barriers. The IMB model has been empirically validated as a framework to identify barriers to diabetes medication adherence [23], but only 1 intervention based on the IMB model has documented its use in Latin American adults with diabetes [35]. Our study supports the use of the IMB framework to better ascertain barriers to diabetes medication adherence among Latino adults, which can be helpful in the development of interventions to address these barriers. Our qualitative analysis also highlights the importance of treatment complexity in this patient population and underscores the need for interventions that can identify and reduce treatment burden and regimen complexity. Although these challenges to diabetes self-management have been well described [36-39], scalable interventions that can reduce the treatment burden are lacking, particularly in the Latino population.

Digital health technology can improve diabetes medication adherence behavior [21,40] and HbA1c [21,40,41] and is recommended by the American Diabetes Association to support diabetes self-care [42]. However, among the 90 (approximately) digital health apps currently available for diabetes self-management, only one-third are available in Spanish, of which 94% (90/96) have a readability level above that recommended for patient education material [43]. Some digital medication adherence support platforms are currently under study in Latino and Spanish-speaking populations [44,45]. However, none of these platforms provide educational content tailored to patient-reported barriers to diabetes medication adherence. The REACH-Español platform adapted in this study offers this key innovation, in addition to health literacy–informed content, which could facilitate broader engagement of prospective participants and long-term medication adherence.

Our study has the key strength of having adapted an intervention with iterative input from stakeholders who could be representative of the population most likely to use the intervention. This user-centered approach is recommended in diabetes digital health research [46,47] to ensure that end user perspectives are represented in the development of interventions and content is tailored to user needs. However, our study also has several limitations. First, the sample size was relatively small, although we achieved thematic saturation for barrier selection after conducting 3 focus groups. Second, we recruited participants from a single health center and did not have representation from every Latin American and Caribbean country, which could limit the generalizability of our findings. In addition, although the barriers to diabetes medication adherence selected in this study were based on relevance to the focus group participants, the order of relevance may differ in other Latin American populations living elsewhere in the United States or with a different insulin usage profile. For instance, “I have trouble paying for my medicine” was not a highly ranked barrier but this may be an important barrier in other areas of the country with lower insurance coverage. Thus, should the REACH-Español content be adapted to other contexts, using the IMB model to identify population-specific barriers could be helpful. Finally, the focus of this study was to adapt the barrier-specific text message content. While text message content pertaining to diet and physical activity was directly translated from English to Spanish, the content did not undergo the stakeholder-driven adaptation process described here, which will be the subject of a future study.

### Conclusions

In this study, we report the multiphase, stakeholder-driven adaptation of the REACH digital health platform text message content, which addresses barriers to diabetes medication adherence, to a Latino population with T2D. We found key barriers to diabetes medication adherence that were relevant to this population, largely related to information gaps. These findings can contribute to the fields of patient-important outcomes and treatment burden to guide respectful and meaningful patient engagement in the management of T2D. Furthermore, engaging a diverse group of bilingual stakeholders (bilingual research team, focus group participants) early and throughout the iterative adaptation process was integral to ensuring content and linguistic relevance of the text messages in the REACH-Español platform. The feasibility, acceptability, usability, and preliminary efficacy of REACH-Español is under study in a 6-month pilot randomized controlled trial, which could broaden our understanding of the role of tailored, digital health interventions on improving diabetes self-care among Latino adults with T2D.

## Supplementary material

10.2196/66668 Multimedia Appendix 1 List of REACH barriers and additional barriers from literature search of potential relevance to Spanish-speaking adults with type 2 diabetes (table shows the barriers to diabetes medication adherence used in the original REACH intervention, mapped into the Information Motivation Behavioral skills model; additional barriers with described relevance to Spanish-speaking adults with type 2 diabetes are shown in gray).

10.2196/66668 Multimedia Appendix 2 Condensed list of barriers used in focus groups 1-3 (table shows the barriers to diabetes medication adherence used in the adaptation of REACH to REACH-Español. The 9 barriers that were identified in the literature with potential relevance for Latino adults with type 2 diabetes were added to the list and combined with similar REACH barriers [gray]).

10.2196/66668 Multimedia Appendix 3 Barrier ranking exercise. Top: “Traffic light” visual tool used to illustrate the ranking categories and corresponding folders used in the ranking exercise. Bottom: Barriers to diabetes medication adherence cards used in the ranking exercise.

10.2196/66668 Multimedia Appendix 4 Barriers to diabetes medication adherence assessed in focus groups conducted with Latino adults with type 2 diabetes and corresponding proportion of participants who reported that the barrier was relevant sometimes or always.

10.2196/66668 Multimedia Appendix 5 Focus group activity to elicit participant input regarding text message content and language. Yes (Si) and No banners were used by participants to prompt discussion on text message content and language.

10.2196/66668 Multimedia Appendix 6 Facilitators to diabetes medication adherence based on thematic content analysis.

## Abbreviations

BRIEF: Brief Health Literacy Screening Tool
HBA_1c_: glycated hemoglobin
IMB: information-motivation-behavioral
MGH: Massachusetts General Hospital
REACH: Rapid Encouragement/Education And Communications for Health
T2D: type 2 diabetes
