# Supplementary material for: Adapting a Text Messaging Intervention to Improve Diabetes Medication Adherence in a Spanish-Speaking Population: Qualitative Study
Source: JMIR Hum Factors. 2025 May 1;12:e66668. doi: 10.2196/66668 (PMC12061353; doi:10.2196/66668)
Supplement: Multimedia Appendix 1 [file humanfactors-v12-e66668-s001.docx]

| **IMB domain** | **Barrier** |
| --- | --- |
| **Information** | I think brand name medicine works better than generic medicine |
|  | I’m disappointed when my medicine doesn’t improve my diabetes right away |
|  | I’m not sure what my diabetes medicine is supposed to do |
|  | I think taking medicine won’t help control my blood sugars or prevent me from having complications |
|  | I’m not sure why my doctor sometimes changes my dose or type of medicine |
|  | I think it is OK to skip or stop taking my medicine on my own |
|  | I think diabetes medicine is not important when I feel well |
|  | I think medicine isn’t important for managing diabetes |
|  | I don’t understand why I need to take a medication that lowers the sugar level |
| **Personal Motivation** | I feel burned out with having to take diabetes medicines |
|  | I worry that taking diabetes medicines for a long time will be bad for me |
|  | I’m afraid of experiencing a side effect from my diabetes medicine |
|  | I believe diabetes medication can be harmful |
|  | I believe my health will get worse no matter how often I take my medicine |
|  | I am afraid of the side effects of taking insulin |
|  | My diabetes medicine is unpleasant to take |
|  | I worry that taking diabetes medicines will cause me to gain weight |
|  | I prefer to take natural medications for my diabetes |
|  | I know people who have died after taking insulin |
|  | I believe that taking insulin can worsen my diabetes |
|  | I believe that taking insulin can cause damage to my organs |
| **Social Motivation** | Juggling other responsibilities makes medicine difficult |
|  | Friends and family nag and annoy me about remembering to take my medicine |
|  | I am embarrassed to take my insulin in front of other people |
|  | When my family or friends remind me to take my medicine, it makes me feel like a child |
|  | I feel embarrassed when taking medicine in front of others |
|  | I worry that people judge me because I take insulin |
|  | I feel others judge me for taking diabetes medicine |
|  | The people I care about don’t support my efforts to take my diabetes medicines |
|  | People close to me say taking my medicine isn’t important |
|  | Family or friends say I shouldn’t take diabetes medicine |
| **Behavioral Skills** | I forget to take my medicine |
|  | I have problems with pain when injecting insulin |
|  | I have trouble paying for medicine |
|  | I forget to order refills |
|  | I have trouble picking up refills |
|  | I have trouble reading medicine labels |
|  | Taking insulin disrupts my daily activities |
|  | Taking diabetes medicine disrupts my daily activities |
|  | My daily medicine routine is too complicated to keep track of |
|  | It is hard for me to ask my doctor about problems with my diabetes medicine |
|  | I don’t understand my doctor’s instructions (in English), even with use of an interpreter |
|  | I don’t understand the instructions in my prescriptions (not written in Spanish) |
|  | When I leave a clinic visit, I am confused as to which medications I need to take |
|  | I don’t know how to ask for refills when the provider does not speak English |
