# Supplementary material for: Adapting a Text Messaging Intervention to Improve Diabetes Medication Adherence in a Spanish-Speaking Population: Qualitative Study
Source: JMIR Hum Factors. 2025 May 1;12:e66668. doi: 10.2196/66668 (PMC12061353; doi:10.2196/66668)
Supplement: Multimedia Appendix 2 [file humanfactors-v12-e66668-s002.docx]

| **Barrier #** | **Barrier content (English)** | **Barrier content (Spanish)** |
| --- | --- | --- |
| 1 | I’m not sure what my diabetes medicine is supposed to do. For example, I don’t understand why I need to take a medication that lowers the sugar level | No estoy seguro de lo que se supone que hace mi medicamento para la diabetes. Por ejemplo, no entiendo por qué tengo que tomar un medicamento que reduce el nivel de azúcar. |
| 2 | I think taking medicine won’t help control my blood sugars or prevent me from having complications | Creo que tomar el medicamento no me ayudará a controlar mis niveles de azúcar en sangre ni a evitar que tenga complicaciones |
| 3 | I’m not sure why my doctor sometimes changes my dose or type of medicine | No estoy seguro de por qué mi médico a veces cambia mi dosis o el tipo de medicamento |
| 4 | I think it is OK to skip or stop taking my medicine on my own | Creo que está bien saltarse o dejar de tomar mi medicamento por mi cuenta |
| 5 | I think diabetes medicine is not important when I feel well | Creo que el medicamento para la diabetes no es importante cuando me siento bien |
| 6 | I think medicine isn’t important for managing diabetes | Creo que el medicamento no es importante para controlar la diabetes |
| 7 | I prefer to take natural medications for my diabetes | Prefiero tomar medicamentos naturales para mi diabetes |
| 8 | I believe diabetes medication can be harmful. For example, I know people who have died after taking insulin | Creo que el medicamento para la diabetes puede ser perjudicial. Por ejemplo, conozco a personas que han muerto después de administrarse insulina |
| 9 | I am afraid of the side effects of taking insulin. For example, I believe that taking insulin can worsen my diabetes | Tengo miedo de los efectos secundarios de la administración de insulina. Por ejemplo, creo que administrarme insulina puede empeorar mi diabetes |
| 10 | I am afraid of the side effects of taking insulin. For example, I believe that taking insulin can cause damage to my organs | Tengo miedo de los efectos secundarios de la administración de insulina. Por ejemplo, creo que administrarme insulina puede dañar mis órganos. |
| 11 | When my family or friends remind me to take my medicine, it makes me feel like a child | Cuando mi familia o mis amigos me recuerdan que debo tomar mi medicamento, me hacen sentir como un niño |
| 12 | I feel embarrassed when taking medicine in front of others | Me siento avergonzado cuando tomo el medicamento delante de los demás |
| 13 | I worry that people judge me because I take insulin | Me preocupa que la gente me juzgue porque me inyecto insulina |
| 14 | I feel others judge me for taking diabetes medicine | Siento que los demás me juzgan por tomar el medicamento para la diabetes |
| 15 | The people I care about don’t support my efforts to take my diabetes medicines | Las personas que me importan no apoyan mis esfuerzos de tomar mis medicamentos para la diabetes |
| 16 | People close to me say taking my medicine isn’t important | Las personas que me rodean dicen que tomar mi medicamento no es importante |
| 17 | Family or friends say I shouldn’t take diabetes medicine | Mi familia o mis amigos dicen que no debo tomar el medicamento para la diabetes |
| 18 | I have trouble reading medicine labels | Tengo problemas para leer las etiquetas del medicamento |
| 19 | Taking diabetes medicine disrupts my daily activities | Tomar el medicamento para la diabetes altera mis actividades diarias |
| 20 | My daily medicine routine is too complicated to keep track of | Mi rutina diaria respecto al medicamento es demasiado complicada de seguir |
| 21 | It is hard for me to ask my doctor about problems with my diabetes medicine. For example, I don’t know how to ask for refills when the provider does not speak English | Me resulta difícil consultarle a mi médico los problemas relacionados con mi medicamento para la diabetes. Por ejemplo, no sé cómo pedir mas medicamentos cuando el proveedor no habla español. |
| 22 | I don’t understand my doctor’s instructions (in English), even with use of an interpreter | No entiendo las instrucciones de mi médico (en inglés), ni siquiera con la ayuda de un intérprete |
| 23 | I don’t understand the instructions in my prescriptions (not written in Spanish) | No entiendo las instrucciones de mis prescripciones (que no están escritas en español) |
| 24 | When I leave a clinic visit, I am confused as to which medications I need to take | Cuando salgo de una visita a la clínica, estoy confundido sobre los medicamentos que tengo que tomar |
| 25 | I think brand name medicine works better than generic medicine | Creo que el medicamento de marca actúa mejor que el genérico |
| 26 | I’m disappointed when my medicine doesn’t improve my diabetes right away | Me siento decepcionado cuando mi medicamento no mejora mi diabetes de inmediato |
| 27 | I feel burned out with having to take diabetes medicines | Me siento agotado por tener que tomar medicamentos para la diabetes |
| 28 | I worry that taking diabetes medicines for a long time will be bad for me | Me preocupa que tomar medicamentos para la diabetes durante mucho tiempo sea malo para mí |
| 29 | I believe my health will get worse no matter how often I take my medicine | Creo que mi salud empeorará sin importar con qué frecuencia tome mi medicamento |
| 30 | My diabetes medicine is unpleasant to take | Mi medicamento para la diabetes es desagradable de tomar |
| 31 | I worry that taking diabetes medicines will cause me to gain weight | Me preocupa que tomar medicamentos para la diabetes me haga aumentar de peso |
| 32 | Juggling other responsibilities makes medicine difficult | Hacer malabares con otras responsabilidades dificulta que tome mi medicamento |
| 33 | Friends and family nag and annoy me about remembering to take my medicine | Mis amigos y mi familia me insisten y me fastidian para que me acuerde de tomar mi medicamento |
| 34 | I am embarrassed to take my insulin in front of other people | Me da vergüenza administrarme insulina delante de otras personas |
| 35 | I forget to take my medicine | Me olvido de tomar mi medicamento |
| 36 | I have problems with pain when injecting insulin | Siento dolor al inyectarme insulina |
| 37 | I have trouble paying for medicine | Tengo problemas para pagar el medicamento |
| 38 | I forget to order refills | Me olvido de pedir reposiciones/ prescripciones |
| 39 | I have trouble picking up refills | Tengo problemas para recoger las reposiciones/ prescripciones |
| 40 | Taking insulin disrupts my daily activities | La administración de insulina altera mis actividades diarias |
