# Supplementary figures and images for: Adapting a Text Messaging Intervention to Improve Diabetes Medication Adherence in a Spanish-Speaking Population: Qualitative Study
Source: JMIR Hum Factors. 2025 May 1;12:e66668. doi: 10.2196/66668 (PMC12061353; doi:10.2196/66668)

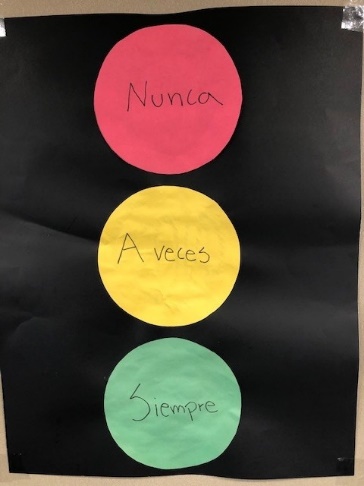


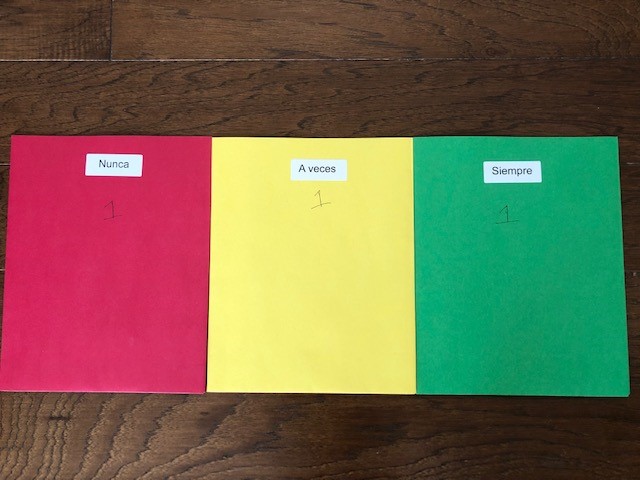


Never

Sometimes

Always


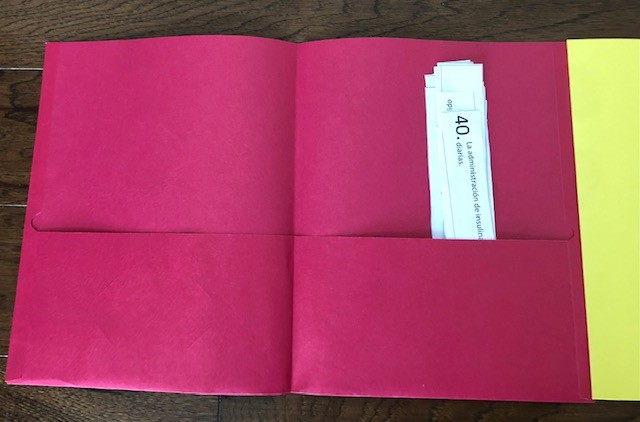

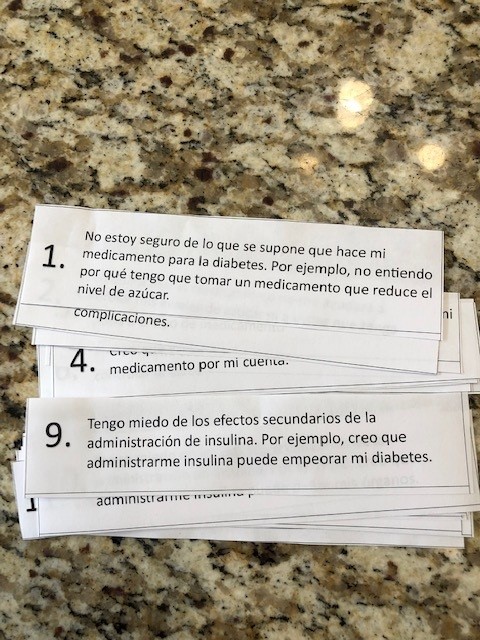

Supplement: Multimedia Appendix 3 [file humanfactors-v12-e66668-s003.docx]

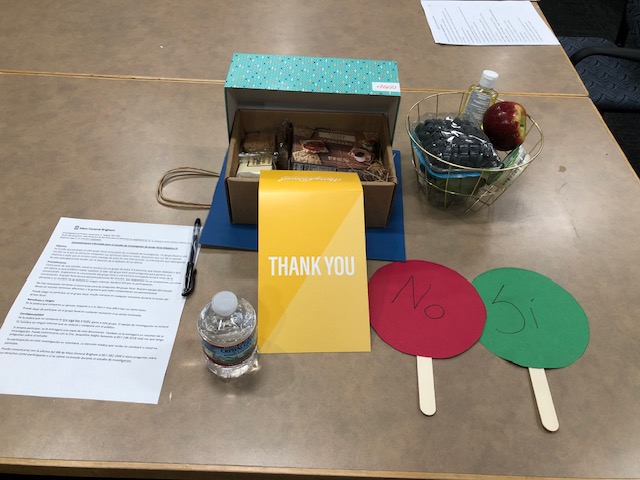

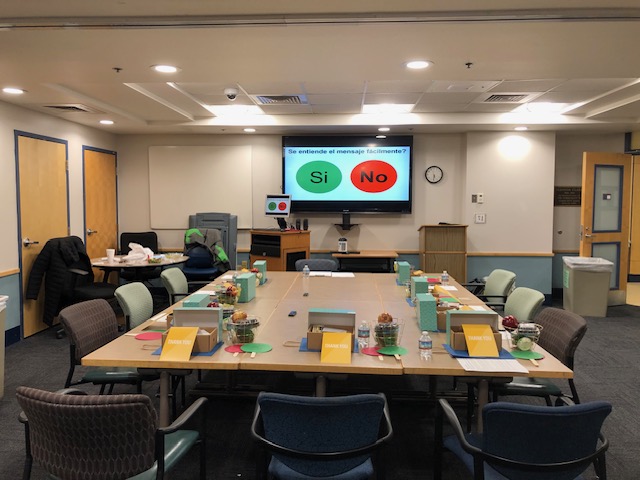

Supplement: Multimedia Appendix 5 [file humanfactors-v12-e66668-s005.docx]
