# Supplementary material for: Adapting a Text Messaging Intervention to Improve Diabetes Medication Adherence in a Spanish-Speaking Population: Qualitative Study
Source: JMIR Hum Factors. 2025 May 1;12:e66668. doi: 10.2196/66668 (PMC12061353; doi:10.2196/66668)
Supplement: Multimedia Appendix 4 [file humanfactors-v12-e66668-s004.docx]

| **REACH-Es Barrier (Spanish)** | **REACH-ES Barrier (English)** | **Proportion of participants reporting barrier sometimes or always** |
| --- | --- | --- |
| No estoy seguro de lo que se supone que hace mi medicamento para la diabetes. Por ejemplo, no entiendo por qué tengo que tomar un medicamento que reduce el nivel de azúcar. | I’m not sure what my diabetes medicine is supposed to do. For example, I don’t understand why I need to take a medication that lowers the sugar level | 61 |
| Creo que el medicamento de marca actúa mejor que el genérico | I think brand name medicine works better than generic medicine | 61 |
| Creo que tomar el medicamento no me ayudará a controlar mis niveles de azúcar en sangre ni a evitar que tenga complicaciones | I think taking medicine won’t help control my blood sugars or prevent me from having complications | 50 |
| No estoy seguro de por qué mi médico a veces cambia mi dosis o el tipo de medicamento | I’m not sure why my doctor sometimes changes my dose or type of medicine | 50 |
| Cuando mi familia o mis amigos me recuerdan que debo tomar mi medicamento, me hacen sentir como un niño | When my family or friends remind me to take my medicine, it makes me feel like a child | 50 |
| Me preocupa que tomar medicamentos para la diabetes durante mucho tiempo sea malo para mí | I worry that taking diabetes medicines for a long time will be bad for me | 50 |
| Hacer malabares con otras responsabilidades dificulta que tome mi medicamento | Juggling other responsibilities makes medicine difficult | 50 |
| Tengo problemas para leer las etiquetas del medicamento | I have trouble reading medicine labels | 44 |
| Me siento decepcionado cuando mi medicamento no mejora mi diabetes de inmediato | I’m disappointed when my medicine doesn’t improve my diabetes right away | 44 |
| Me olvido de tomar mi medicamento | I forget to take my medicine | 44 |
| Siento dolor al inyectarme insulina | I have problems with pain when injecting insulin | 44 |
| Siento que los demás me juzgan por tomar el medicamento para la diabetes | I feel others judge me for taking diabetes medicine | 39 |
| No entiendo las instrucciones de mis prescripciones (que no están escritas en español) | I don’t understand the instructions in my prescriptions (not written in Spanish) | 39 |
| Mi medicamento para la diabetes es desagradable de tomar | My diabetes medicine is unpleasant to take | 39 |
| Me da vergüenza administrarme insulina delante de otras personas | I am embarrassed to take my insulin in front of other people | 39 |
| Me olvido de pedir reposiciones/ prescripciones | I forget to order refills | 39 |
| Tengo problemas para recoger las reposiciones/ prescripciones | I have trouble picking up refills | 39 |
| Tengo miedo de los efectos secundarios de la administración de insulina. Por ejemplo, creo que administrarme insulina puede empeorar mi diabetes | I am afraid of the side effects of taking insulin. For example, I believe that taking insulin can worsen my diabetes | 33 |
| Tengo miedo de los efectos secundarios de la administración de insulina. Por ejemplo, creo que administrarme insulina puede dañar mis órganos. | I am afraid of the side effects of taking insulin. For example, I believe that taking insulin can cause damage to my organs | 33 |
| Me siento agotado por tener que tomar medicamentos para la diabetes | I feel burned out with having to take diabetes medicines | 33 |
| Creo que mi salud empeorará sin importar con qué frecuencia tome mi medicamento | I believe my health will get worse no matter how often I take my medicine | 33 |
| Me preocupa que tomar medicamentos para la diabetes me haga aumentar de peso | I worry that taking diabetes medicines will cause me to gain weight | 33 |
| Mis amigos y mi familia me insisten y me fastidian para que me acuerde de tomar mi medicamento | Friends and family nag and annoy me about remembering to take my medicine | 33 |
| Las personas que me importan no apoyan mis esfuerzos de tomar mis medicamentos para la diabetes | The people I care about don’t support my efforts to take my diabetes medicines | 28 |
| Las personas que me rodean dicen que tomar mi medicamento no es importante | People close to me say taking my medicine isn’t important | 28 |
| Tomar el medicamento para la diabetes altera mis actividades diarias | Taking diabetes medicine disrupts my daily activities | 28 |
| Mi rutina diaria respecto al medicamento es demasiado complicada de seguir | My daily medicine routine is too complicated to keep track of | 28 |
| Creo que el medicamento no es importante para controlar la diabetes | I think medicine isn’t important for managing diabetes | 22 |
| Prefiero tomar medicamentos naturales para mi diabetes | I prefer to take natural medications for my diabetes | 22 |
| Creo que el medicamento para la diabetes no es importante cuando me siento bien | I think diabetes medicine is not important when I feel well | 17 |
| Creo que el medicamento para la diabetes puede ser perjudicial. Por ejemplo, conozco a personas que han muerto después de administrarse insulina | I believe diabetes medication can be harmful. For example, I know people who have died after taking insulin | 17 |
| Me preocupa que la gente me juzgue porque me inyecto insulina | I worry that people judge me because I take insulin | 17 |
| No entiendo las instrucciones de mi médico (en inglés), ni siquiera con la ayuda de un intérprete | I don’t understand my doctor’s instructions (in English), even with use of an interpreter | 17 |
| Cuando salgo de una visita a la clínica, estoy confundido sobre los medicamentos que tengo que tomar | When I leave a clinic visit, I am confused as to which medications I need to take | 17 |
| Me siento avergonzado cuando tomo el medicamento delante de los demás | I feel embarrassed when taking medicine in front of others | 11 |
| Mi familia o mis amigos dicen que no debo tomar el medicamento para la diabetes | Family or friends say I shouldn’t take diabetes medicine | 11 |
| Tengo problemas para pagar el medicamento | I have trouble paying for medicine | 11 |
| La administración de insulina altera mis actividades diarias | Taking insulin disrupts my daily activities | 11 |
| Creo que está bien saltarse o dejar de tomar mi medicamento por mi cuenta | I think it is OK to skip or stop taking my medicine on my own | 6 |
| Me resulta difícil consultarle a mi médico los problemas relacionados con mi medicamento para la diabetes. Por ejemplo, no sé cómo pedir más medicamentos cuando el proveedor no habla español. | It is hard for me to ask my doctor about problems with my diabetes medicine. For example, I don’t know how to ask for refills when the provider does not speak English | 6 |
|  | Mean (SD) | 32% (15) |
