# Supplementary material for: Adapting a Text Messaging Intervention to Improve Diabetes Medication Adherence in a Spanish-Speaking Population: Qualitative Study
Source: JMIR Hum Factors. 2025 May 1;12:e66668. doi: 10.2196/66668 (PMC12061353; doi:10.2196/66668)
Supplement: Multimedia Appendix 6 [file humanfactors-v12-e66668-s006.docx]

| **Facilitators to diabetes medication adherence: What motivates you to take your diabetes medications?** | |
| --- | --- |
| **Themes** | **Participant Quotes** |
| ***Fear as motivation*** | |
| *Fear of organ damage* | *“Yes, the fear of complications.” (Female, 62 years old, El Salvador)*  *“[When seeing high blood sugars] I think first about my kidneys...”(Female, 62 years old, El Salvador)* |
| ***Belief in treatment benefit*** | |
| *Seeing improvement in blood sugars or weight loss* | *“[Seeing good numbers] Motivates me.” (Female, 62 years old, El Salvador)*  *“I do think it’s helping me to lose weight.” (Male, 65 years old, Guatemala)* |
| *Want to see improvement and live longer* | *“Well, wanting to live a little bit more… (Male, 65 years old, Guatemala).”*  *“In my case, I am sad when I look in the morning and I am [the sugar] is high, so while fasting I say this is too high, this can’t be, so I try to find a way to improve.” (Female, 62 years old, El Salvador)* |
| *Belief that medications are good* | *“Because you know that it is good for you, for one’s health.” (Male, 65 years old, Guatemala)* |
| ***Personal responsibility and will to take medications*** | |
|  | *“As I am telling you, we are adults and have to take personal responsibility. There is no other adult that will tell you take your pill, take your pill, as to a little child.” (Male, 54 years old, El Salvador)*  *“The medication is something that becomes routine. Routine and a responsibility because you know that if you don’t take the medication you will feel the damage.” (Male, 54 years old, El Salvador)*  *“Strong will [to take medications].”(Male, 56 years old, Guatemala).”* |
| ***Family support and medication organization*** | |
| *Reminders by family members* | *“My husband tells me at night, did you take your medicine? Because otherwise, I forget.” (Female, 71 years old, Puerto Rico)*  *“I have to take the medication every Tuesday…if not, my wife reminds me. Starting on Sunday, I ask her to remind me.” (Male, 77 years old, Guatemala)* |
| *Medication organizers* | *“On Sundays, my husband puts all the medications I need to take in a pill box.”(Female, 71 years old, Puerto Rico).”* |
| ***Getting used to the medication*** | |
|  | *“In terms of side effects, it’s possible that your body needs more time to adapt to the medication because I had that experience.” (Female, 53 years old, Honduras)*  *“At first yes [side effects] but when I adapted, not anymore.” (Female, 57 years old, El Salvador)* |
